# Reductions in liver enzymes are associated with anti‐hyperglycaemic and anti‐obesity effects of tofogliflozin in people with type 2 diabetes: Post‐hoc analyses

**DOI:** 10.1002/edm2.461

**Published:** 2023-11-20

**Authors:** Toshinari Takamura, Kohei Kaku, Akihiro Yoshida, Hiromi Kusakabe, Hiroyuki Nakamura, Hideki Suganami

**Affiliations:** ^1^ Department of Endocrinology and Metabolism Kanazawa University Graduate School of Medical Sciences Kanazawa Japan; ^2^ Department of Internal Medicine Kawasaki Medical School Kurashiki Japan; ^3^ Kowa Co., Ltd. Tokyo Japan; ^4^ Department of Hygiene and Public Health , Faculty of Medicine, Institute of Medical, Pharmaceutical and Health Sciences, Kanazawa University Kanazawa Japan; ^5^ Clinical Data Science Department Kowa Co., Ltd. Tokyo Japan

**Keywords:** alanine aminotransferase, fatty liver, gamma‐glutamyltransferase, sodium/glucose cotransporter 2 inhibitor, tofogliflozin

## Abstract

**Aims:**

How the pathology of type 2 diabetes (T2D), including hyperglycaemia and obesity, affects liver enzymes has not been clinically demonstrated. Thus, we compared time courses of gamma‐glutamyltransferase (GGT) and alanine aminotransferase (ALT) with those of fasting plasma glucose (FPG) and body weight (BW) during treatment with the SGLT2 inhibitor tofogliflozin for T2D.

**Materials and Methods:**

We post‐hoc analysed preexisting data on 1046 people with T2D administered tofogliflozin or placebo for 24 weeks in four tofogliflozin studies. First, time courses of percent changes in variables during the intervention were analysed using a mixed effect model to explore the similarity of the time courses and to evaluate time‐treatment interactions. Second, clinical factors related to the percent changes in GGT and ALT were clarified using multivariate analyses.

**Results:**

GGT levels and FPG values rapidly and significantly decreased via tofogliflozin as early as week 4, with decreases maintained until week 24. Conversely, BW and ALT decreased progressively until week 24. Time courses of FPG (*p* = .365, time‐treatment interaction) and GGT (*p* = .510) reductions were parallel between tofogliflozin and placebo from weeks 4 to 24, while BW and ALT reductions (*p* < .001, respectively) were not. Reductions in GGT at week 24 were associated with reductions in FPG and BW at week 24, whereas ALT reductions were only associated with reductions in BW.

**Conclusions:**

Reductions in GGT and ALT were associated with the anti‐hyperglycaemic and anti‐obesity effects of tofogliflozin, respectively, in people with T2D. Therefore, GGT and ALT may be surrogate markers for hyperglycaemia and obesity in T2D.

## Graphical abstract

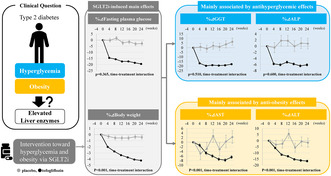

## 1 INTRODUCTION

Nonalcoholic fatty liver disease (NAFLD) is a consequence and cause of insulin resistance and is a common pathology resulting from obesity and hyperglycaemia in type 2 diabetes (T2D).^1^ People with T2D are particularly susceptible to more severe forms of NAFLD.^2^, ^3^, ^4^ Circulating levels of liver enzymes, including those for serum alanine aminotransferase (ALT) and gamma‐glutamyltransferase (GGT), provide insights into the extent of hepatic fat accumulation and its related oxidative stress. ALT is the most specific marker of liver pathology and fat accumulation. Changes in ALT levels were correlated with hepatic fat levels measured by proton spectroscopy after weight loss.^5^ Furthermore, high ALT^6^, ^7^, ^8^, ^9^, ^10^ and GGT^8^, ^10^ levels were identified as significant risk factors for T2D. In a Japanese prospective cohort study, both ALT and GGT were identified as independent predictors of T2D after patients were stratified according to various risk factors, specifically fasting insulin, body mass index (BMI), waist‐hip ratio, and high‐sensitivity C‐reactive protein values.^10^ These findings suggest that elevated liver enzymes values, particularly those for GGT and ALT, indicate the presence of metabolic abnormalities initiated by fat accumulation through weight gain and hyperglycaemia. Additionally, increased GGT and ALT levels were reported to be independent and additive risk factors for the development of T2D in people without fatty liver, suggesting that their increase may be individually reflected by the distinct pathology of T2D.^11^ However, the time‐course of the elevation of each liver enzyme and its specific associations with hyperglycaemia and obesity have not been demonstrated clinically. To address this issue, careful observation of the time‐course of liver enzymes values and a statistical evaluation of similarity of changes in GGT and ALT levels during interventions for hyperglycaemia and obesity may be useful.

A new class of antidiabetic agents, sodium/glucose cotransporter‐2 (SGLT2) inhibitors, reduces both hyperglycaemia and body weight (BW),^12^, ^13^ thereby lowering liver enzyme levels.^14^, ^15^ In the present study, we examined the time course of GGT and ALT levels during interventions for hyperglycaemia and obesity with the SGLT2 inhibitor, tofogliflozin. Further, we explored similarities of time courses of percent changes in variables and evaluated the associations of fasting plasma glucose (FPG) and BW with GGT and ALT in patients with T2D.

## 2 MATERIALS AND METHODS

### 2.1 Integrated studies and patients

The pooled post‐hoc analysis included four phase 2 and phase 3 tofogliflozin trials (Tables S1 and S2) with durations of at least 24 weeks that included people with T2D and compared placebo to different doses of tofogliflozin or compared various doses of tofogliflozin either as monotherapy or as an add‐on to other antidiabetic agents. The CSG003JP study (placebo or tofogliflozin [10, 20, and 40 mg] as monotherapy) was a 24‐week randomized, double‐blind, placebo‐controlled, combined phase 2 and 3 trial.^14^ The CSG004JP study (tofogliflozin 20 and 40 mg as monotherapy) and the CSG005JP study (tofogliflozin 20 and 40 mg as add‐ons to other oral antidiabetic agents) were both 52‐week randomized‐controlled, open‐label, phase 3 trials.^16^ Details of these studies and their results, along with patient inclusion and exclusion criteria, were previously reported.^14^, ^16^ The CSG006JP study (tofogliflozin 40 mg as monotherapy or an add‐on to sulfonylurea or dipeptidyl peptidase‐4 inhibitor) was a 24‐week, multicentre, open‐label trial.^17^ Individual‐level data from the 24‐ or 52‐week core treatment periods of each study were used in this pooled analysis. Details of each study with the main patient inclusion criteria are summarized in Table S1. All included studies were conducted in accordance with the Declaration of Helsinki and Good Clinical Practice. The protocols had been reviewed and approved by the institutional review board of each participating centre (JapicCTI‐101349, JapicCTI‐101351, JapicCTI‐101352 and JapicCTI‐111572). All patients provided written informed consent prior to enrolment in the study. This consent included an agreement for further analysis and publication of the existing data acquired in those studies. Therefore, the requirement for additional informed consent was waved due to the use of preexisting data.

The pooled analysis included 1046 participants who received either tofogliflozin (10, 20 or 40 mg) or placebo (Table S2). Study‐group baseline characteristics were 67% men; mean age, 58 years; glycosylated haemoglobin (HbA1c), 8.1%; FPG, 162 mg/dL; BMI, 26 kg/m^2^; GGT, 48 IU/L; ALT, 29 IU/L; and eGFR, 83 mL/min/1.73 m^2^ (Table 1).

**TABLE 1:** Baseline characteristics of study participants.

*N*	All	BMI < 25 kg/m^2^	BMI≥25 kg/m^2^		FPG <8.7 mmol/L (156 mg/dL)	FPG≥8.7 mmol/L (156 mg/dL)	
	1046	537	509	*p*	521	525	*p*
Age, years	58.3 (10.3)	61.4 (8.6)	55.1 (11.0)	<.001	59.6 (10.4)	57.1 (10.1)	<.0001
Male / Female, *n*	700 / 346	371 / 166	329 / 180	.13	327 / 194	373 / 152	<.01
Placebo / TOFO 10 mg / TOFO 20 mg / TOFO 40 mg, *n*	56 / 57 / 293 / 640	28 / 31 / 147 / 331	28 / 26 / 146 / 309	–	19 / 18 / 150 / 334	37 / 39 / 143 / 306	–
Concomitant antidiabetic drug, *n* (%)	604 (57.7)	313 (58.3)	291 (57.2)	.75	305 (58.5)	299 (57.0)	.62
Monotherapy / SU / glinide / BG / TZD / a‐GI / DPP‐4i, *n*	442 / 180 / 22 / 99 / 99 / 96 / 108	224 / 111 / 13 / 45 / 36 / 55 / 53	218 / 69 / 9 / 54 / 63 / 41 / 55	–	216 / 74 / 9 / 59 / 64 / 55 / 44	226 / 106 / 13 / 40 / 35 / 41 / 64	–
Duration of Diabetes, years	6.9 (5.9)	8.0 (6.3)	5.7 (5.1)	<.001	6.3 (5.3)	7.4 (6.4)	<.01
BMI, kg/m^2^	25.6 (4.2)	22.5 (1.7)	28.9 (3.6)	<.001	25.4 (4.1)	25.8 (4.4)	.12
Systolic blood pressure, mmHg	130.0 (14.0)	127.6 (13.9)	132.5 (13.7)	<.001	129.0 (14.4)	131.0 (13.6)	<.05
Diastolic blood pressure, mmHg	77.6 (10.3)	75.4 (10.1)	79.9 (10.0)	<.001	76.4 (10.8)	78.8 (9.7)	<.001
Serum creatinine, mg/dL	0.72 (0.19)	0.72 (0.19)	0.72 (0.19)	.998	0.74 (0.20)	0.71 (0.18)	<.01
eGFR, mL/min/1.73m^2^	83.5 (19.3)	82.4 (18.5)	84.6 (20.1)	.06	80.0 (18.3)	86.9 (19.7)	<.001
Uric acid, mg/dL	5.1 (1.3)	4.9 (1.3)	5.3 (1.3)	<.001	5.2 (1.3)	5.0 (1.3)	<.05
HbA1c, %	8.1 (0.9)	8.0 (0.9)	8.2 (0.9)	<.01	7.6 (0.5)	8.6 (0.9)	<.001
HbA1c, mmol/mol	65.2 (9.8)	64.3 (9.6)	66.1 (9.9)	<.01	59.4 (5.8)	71.0 (9.5)	<.001
FPG, mg/dL	162.5 (35.6)	159.7 (34.6)	165.4 (36.5)	<.05	135.8 (13.5)	188.9 (30.7)	<.001
FPG, mmol/L	9.0 (2.0)	8.9 (1.9)	9.2 (2.0)	<.05	7.5 (0.7)	10.5 (1.7)	<.001
Fasting insulin, μU/mL	8.9 (19.5)	5.8 (3.2)	12.1 (27.2)	<.001	8.0 (5.7)	9.8 (26.9)	.14
HOMA‐β	34.4 (44.5)	24.0 (15.4)	45.3 (59.9)	<.001	40.5 (30.1)	28.3 (54.6)	<.001
HOMA‐IR	3.7 (12.1)	2.3 (1.4)	5.2 (17.2)	<.001	2.7 (2.0)	4.7 (17.0)	<.01
Body weight, kg	68.6 (13.9)	60.1 (8.2)	77.5 (13.0)	<.001	67.1 (13.5)	70.0 (14.1)	<.001
Waist circumference, cm	89.6 (10.4)	82.9 (6.5)	96.7 (8.9)	<.001	88.8 (10.0)	90.5 (10.8)	<.01
AST, IU/L	26.5 (10.8)	24.4 (8,3)	28.7 (12.5)	<.001	26.2 (9.6)	26.7 (11.9)	.44
ALT, IU/L	29.2 (16.5)	23.8 (12.0)	35.0 (18.6)	<.001	27.3 (15.2)	31.1 (17.5)	<.001
GGT, IU/L	47.9 (51.1)	45.4 (60.6)	50.4 (38.4)	.11	41.3 (45.3)	54.4 (55.5)	<.001
ALP, IU/L	242.2 (99.6)	241.1 (71.2)	243.4 (122.8)	.71	231.5 (70.6)	252.8 (120.9)	<.001
BAP, μg/L	15.4 (6.5)	15.6 (6.3)	15.3 (6.7)	.40	15.0 (6.5)	15.8 (6.4)	<.05
NTx, nmol BCE/L	17.1 (6.2)	17.7 (7.1)	16.5 (4.8)	<.01	17.5 (6.7)	16.7 (5.5)	<.05
TC, mg/dL	205.9 (34.0)	207.1 (33.2)	204.6 (34.9)	.25	201.9 (33.7)	209.8 (34.0)	<.001
LDL‐C, mg/dL	124.6 (29.9)	123.9 (29.0)	125.4 (30.9)	.43	122.1 (30.4)	127.1 (29.3)	<.01
HDL‐C, mg/dL	59.4 (16.9)	63.8 (18.4)	54.6 (13.8)	<.001	60.7 (17.5)	58.0 (16.3)	<.05
TG, mg/dL	152.0 (126.9)	134.6 (134.4)	170.4 (115.8)	<.001	132.4 (82.5)	171.5 (156.8)	<.001
β‐hydroxybutyrate μmol/L	75.3 (89.3)	83.5 (102.0)	66.7 (72.8)	<.01	74.9 (94.9)	75.8 (83.5)	.87

*Note*: Data are expressed as mean (SD).

*Note*: Analyses were performed by one‐way analysis of variance and Fisher's exact test.

Abbreviations: ALP, alkaline phosphatase; ALT, alanine aminotransferase; AST, aspartate aminotransferase; BAP, bone specific alkaline phosphatase; BG, biguanide; BMI, body mass index; DPP‐4i, dipeptidyl peptidase‐4 inhibitor; eGFR, estimated glomerular filtration rate; FPG, fasting plasma glucose; GGT, gamma‐glutamyltransferase; HbA1c, glycosylated haemoglobin; HDL‐C, high density lipoprotein‐cholesterol; HOMA‐IR, homeostatic model assessment of insulin resistance; HOMA‐β, homeostatic model assessment of β cell function; LDL‐C, low density lipoprotein‐cholesterol; NTx, type I collagen N‐terminal telopeptide; SU, sulfonylurea; TC, total cholesterol; TG, triglycerides; TOFO, tofogliflozin; TZD, thiazolidine; α‐GI, α‐glucosidase inhibitor.

### 2.2 Measurements

Baseline laboratory values measured for the following variables: HbA1c, FPG, homeostatic model assessment of insulin resistance (HOMA‐IR), homeostatic model assessment of beta‐cell function (HOMA‐beta), liver enzymes (GGT, ALT, aspartate aminotransferase [AST] and alkaline phosphatase [ALP]), bone‐specific alkaline phosphatase (BAP), type I collagen N‐terminal telopeptide (NTx), uric acid, serum lipids (total cholesterol [TC], low‐density lipoprotein‐cholesterol [LDL‐C], high‐density lipoprotein‐cholesterol [HDL‐C], triglycerides [TG]), serum creatinine, and the estimated glomerular filtration rate (eGFR) calculated from serum creatinine values and β‐hydroxybutyrate.

### 2.3 Overall statistical flow in this post hoc analyses

Each study involved in this post‐hoc analysis included a plan for statistical analysis. However, since this is a post hoc analysis of several interventional studies, there was not a plan for the statistical analysis. However, we examined the data as follows. First, we investigated baseline characteristics of variables in the study patients including those of hepatic enzymes to assess the effects of obesity (BMI) and glycemic status (FPG) on them. The associations between hepatic enzymes and variables were then explored using univariate and multivariate analyses. Second, the time‐course of changes during the interventions were investigated to assess over time the associations among the time course of changes in hepatic enzymes, FPG, and BW using the mixed‐effects model for repeated measures approach. Finally, we investigated associations among the changes in hepatic enzymes, FPG, and BW using univariate and multivariate analyses. The two‐sided significance level for each test was 0.05.

### 2.4 Statistical analyses

#### 2.4.1 Evaluation of clinical parameters at baseline

To assess the effects of obesity and glycemic status, respectively, on baseline parameters, participants were divided into two groups according to the presence of obesity (BMI ≥25 kg/m^2^) and baseline median FPG levels (FPG ≥8.7 mmol/L [156 mg/dL]).

Analyses of combinations of correlations among baseline liver enzymes, FPG levels and BMI values were performed using Spearmanʼs rank order correlation coefficients.

To examine whether baseline FPG levels and BMI values might be associated with GGT and ALT baseline levels in the entire participant population, 12 variables at baseline (age, sex, duration of diabetes, HbA1c, FPG, HOMA‐IR, HOMA‐beta, BMI, eGFR, waist circumference, AST, ALT and GGT) were initially identified according to clinical considerations.

#### 2.4.2 Statistical evaluation of the time‐course of variables during the entire intervention

To examine time courses of the following variables, percentage changes in FPG, BW, GGT, ALT, AST, ALP, BAP and NTx during the whole intervention were tested using a mixed effect model with treatment (tofogliflozin vs. placebo), time (FPG, BW, GGT, ALT, AST, and ALP; from week 4 to 24 and BAP, and NTx; week 12 and 24) and interaction between treatment and time as fixed effects, baseline value as a covariate, and subject as a random effect.

#### 2.4.3 Evaluation of clinical parameters that are independently associated and predictive of GGT and ALT reductions

Analyses of correlations among combinations of percentage changes in liver enzymes, FPG, and BW at week 24 in participants receiving tofogliflozin.

To examine whether the percent changes in FPG levels and BW might affect the percent changes in GGT and ALT levels at week 24 in participants receiving tofogliflozin, 13 variables at baseline (age, sex, duration of diabetes, HbA1c, FPG, HOMA‐IR, HOMA‐beta, BMI, eGFR, waist circumference, AST, GGT and ALT) and 2 variables at week 24 (percent changes in FPG levels and BW) were initially identified according to clinical considerations. In addition, one variable at week 24 (percent change in HOMA‐IR) was added to the above used model for further consideration. Additionally, to examine whether the percent changes in FPG levels and BW might affect the those in GGT and ALT levels at week 4, the same procedure was performed using the percentage of changes in variables at week 4 instead of changes at week 24. Finally, to examine whether FPG and BW reductions might predict the percent changes in GGT and ALT levels at week 24, the percent changes in GGT and ALT levels at week 4 were added to the above baseline variables. These factors were identified using a multivariate regression model and stepwise variable selection (p < 0.05).

The participants' demographics were summarized using appropriate descriptive statistics (mean and standard deviation [SD] for continuous variables, numeric values and percentages for categorical variables). Furthermore, differences in assessments across groups were analysed using analysis of variance (ANOVA) and Fisher's exact test. In the present study, all HbA1c values are presented in National Glycohemoglobin Standardization Program (NGSP) units.

## 3 RESULTS

### 3.1 Association of basal GGT and ALT levels with clinical parameters

At baseline, both GGT and ALT levels were positively associated with FPG levels and BMI values (Table S3). Multivariate analyses suggested that GGT levels were positively associated with FPG levels not BMI values and that ALT levels were positively associated with BMI and HbA1c (Table S4). When participants were stratified according to BMI and FPG, basal levels of ALT and AST but not GGT and ALP were significantly higher in participants with a BMI ≥25 kg/m^2^ (Table 1). Also, values for ALT, GGT and ALP but not AST were significantly higher in participants with FPG ≥8.7 mmol/L (156 mg/dL).

### 3.2 Time‐course of percent changes in GGT, ALT, FPG and BW during the intervention with tofogliflozin

FPG levels after the start of tofogliflozin therapy had rapidly and significantly decreased as early as week 4 (least squares mean: −14.4%, *p* < .001 vs. placebo). This decrease was maintained until week 24 (−16.7%, *p* < .001). Similarly, GGT levels had rapidly and significantly decreased at week 4 (−17.8%, *p* < .001), and the decrease was maintained until week 24 (−24.4%, *p* < .001). ALP levels were significantly decreased at week 4 (−5.3%, *p* < .01), which was maintained until week 24 (−6.9%, *p* < .001). On the other hand, BW decreased progressively until week 24 (−3.8%, *p* < .001) as did ALT and AST levels (−15.0%, *p* < .001 and − 8.7%, *p* < .01 respectively).

**FIGURE 1:**
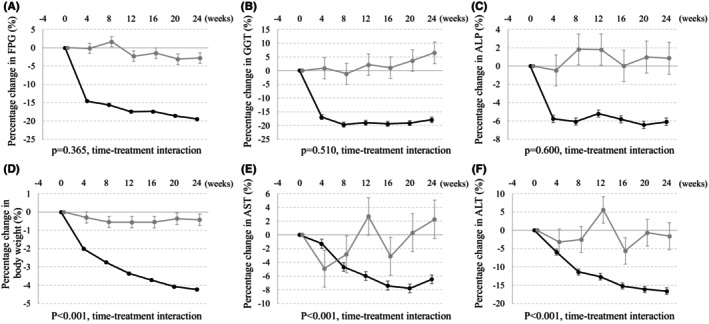
Time‐courses of percentage changes in levels of FPG (A), GGT (B), ALP (C), body weight (D), AST (E), and ALT (F). Observed least square mean (standard error). ● placebo, ● tofogliflozin. The time courses of the percent changes in FPG, GGT, ALP, body weight, AST and ALT during entire intervention were tested using a mixed effect model with treatment (tofogliflozin vs. placebo) and time (from week 4 to 24) and interaction between treatment and time as fixed effects with the baseline value as a covariate, and subject as a random effect. P value for time‐treatment interaction was reported. ALP, alkaline phosphatase; ALT, alanine aminotransferase; AST, aspartate aminotransferase; FPG, fasting plasma glucose; GGT, gamma‐glutamyltransferase.

Time courses of BW and reductions in ALT, AST and NTx were not parallel between tofogliflozin and placebo (*p* < .01, time‐treatment interaction) (Figure 1 and Figure S1), while those of reductions in FPG (*p* = .365), GGT (*p* = .510), ALP (*p* = .600), and BAP (*p* = .852) were parallel (Figure 1 and Figure S1).

### 3.3 Clinical parameters that were associated with and predictive of GGT and ALT reductions at week 24 in participants receiving tofogliflozin

At week 24, reductions in both GGT and ALT were positively associated with reductions in FPG and BW (Table S5).

Because the time courses of these reductions were distinct according to the careful investigation and statistical evaluation, we analysed factors influencing these reductions at weeks 4 and 24 in participants receiving tofogliflozin using a multivariate stepwise method (Table 2, Table 3 and Table 4). The reduction at week 24 in GGT was independently associated with the reductions in FPG and BW at week 24 (Table 2), whereas the reduction at week 24 in ALT was associated with the reduction in only BW but not FPG (Table 2). In addition, we added the percent change in HOMA‐IR at week 24 into the above multivariate model as a factor that might influence the reductions in GGT and ALT at week 24. The reductions of both liver enzymes were independently associated with the reduction in HOMA‐IR at week 24; however, the influences of the reductions in FPG and BW for liver enzymes were maintained (Table S6). Similarly, at week 4, the reduction in GGT was associated with reductions in FPG and BW at week 4 (Table 3), whereas the overall reduction in ALT was not associated with either FPG or BW reductions (Table 3). Finally, the early reductions in FPG and BW at week 4 predicted the reduction in GGT at week 24, whereas a reduction in only BW at week 4 predicted the reduction in ALT (Table 4).

**TABLE 2:** Potential factors influencing percentage of changes in GGT and ALT levels at week 24 in participants receiving tofogliflozin.

Percent change in GGT levels at week 24		
Factors	Regression coefficient	*p*
Percent change in body weight at week 24 (>1%)	1.58	<.001
Percent change in FPG at week 24 (>1%)	0.33	<.001
AST (>1 IU/L)	0.22	<.001
Age (>1 year)	0.08	.017
FPG (>1 mg/dL)	0.06	<.001
HOMA‐β (>1 unit)	0.03	<.001
GGT (>1 IU/L)	−0.08	<.001
ALT (>1 IU/L)	−0.32	<.001
Percent change in ALT levels at week 24		
Factors	Regression coefficient	*p*
Percent change in body weight at week 24 (>1%)	1.13	<.001
AST (>1 IU/L)	0.17	.001
HOMA‐IR (>1 unit)	0.07	.008
eGFR (>1 mL/min/1.73 m^2^)	0.04	.036
ALT (>1 IU/L)	−0.60	<.001

*Note*: Stepwise variable selection was used for the analyses.

Adjusted for age, sex, duration of diabetes, HbA1c, FPG, HOMA‐IR, HOMA‐β, BMI, eGFR, waist circumference, AST, ALT, GGT, the percentage change in FPG at week 24 and the percent change in body weight at week 24.

**TABLE 3:** Potential factors influencing percentage of changes in GGT and ALT levels at week 4 in participants receiving tofogliflozin.

Percent change in GGT levels at week 4		
Factors	Regression coefficient	*p*
Percent change in body weight at week 4 (>1%)	2.09	<.001
Percent change in FPG at week 4 (>1%)	0.33	<.001
GGT (>1 IU/L)	−0.08	<.001
Percent change in ALT levels at week 4		
Factors	Regression coefficient	*p*
BMI (>1 kg/m^2^)	0.70	.002
ALT (>1 IU/L)	−0.44	<.001

*Note*: Stepwise variable selection was used for the analyses.

Adjusted for age, sex, duration of diabetes, HbA1c, FPG, HOMA‐IR, HOMA‐β, BMI, eGFR, waist circumference, AST, ALT, GGT, the percentage change in FPG at week 4 and the percent change in body weight at week 4.

**TABLE 4:** Potential predictors influencing percentage of changes in GGT and ALT levels at week 24 in participants receiving tofogliflozin.

Percent change in GGT levels at week 24		
Predictors	Regression coefficient	*p*
Percent change in body weight at week 4 (>1%)	1.80	<.001
BMI (>1 kg/m^2^)	0.25	.003
AST (>1 IU/L)	0.22	<.001
Duration of diabetes (>1 year)	0.19	<.001
Percent change in FPG at week 4 (>1%)	0.17	<.001
GGT (>1 IU/L)	−0.08	<.001
ALT (>1 IU/L)	−0.33	<.001
Percent change in ALT levels at week 24		
Predictors	Regression coefficient	*p*
Percent change in body weight at week 4 (greater 1%)	1.09	<.001
AST (>1 IU/L)	0.14	.005
eGFR (>1 mL/min/1.73 m^2^)	0.02	.131
ALT (>1 IU/L)	−0.58	<.001

*Note*: Stepwise variable selection was used for the analyses.

Adjusted for age, sex, duration of diabetes, HbA1c, FPG, HOMA‐IR, HOMA‐β, BMI, eGFR, waist circumference, AST, ALT, GGT, the percentage change in FPG at week 4 and the percent change in body weight at week 4.

## 4 DISCUSSION

Although the ductal enzyme GGT and liver enzyme ALT are used clinically as severity indices of fatty liver disease, the mechanisms and time‐course of their elevations remain unclear. The present study proved with the mixed‐effects model for repeated measure approach that time courses of the decreases in GGT and ALT levels during the intervention to treat hyperglycaemia and obesity with the SGLT2 inhibitor, tofogliflozin, are distinct from each other. Also, the time courses of decreases in FPG levels and BW were distinct. Multivariate analyses indicated associations of BW reductions with both GGT and ALT reductions. However, FPG reductions were associated with a greater reduction in GGT but not ALT. These findings indicate that GGT and ALT reflect distinct pathologies of T2D, that is, glycemic status and BW, respectively. GGT but not ALT reflects glycemic status whereas both enzymes reflect obesity in people with T2D.

GGT is a ubiquitous cell membrane and plasma‐circulating enzyme involved in the cellular metabolism of glutathione (GSH). As GSH is a major antioxidant, GGT is considered a protective enzyme, contributing to the maintenance of the cellular redox status.^18^, ^19^ Indeed, it was reported that an elevated serum GGT level might be used to identify people with increased oxidative stress.^20^, ^21^, ^22^ Hyperglycaemia leads to the generation of reactive oxygen species via NADPH oxidases.^23^ Based on these findings, we speculate that in the present study, GGT levels decreased during the intervention with tofogliflozin mainly via a reduction in hyperglycaemia‐associated oxidative stress, which should be investigated in the future.

In the present study, baseline ALP levels were significantly higher in the participants with higher FPG levels but did not differ between those with and without obesity (Table 1). ALP reduction was significantly correlated with FPG reduction, but not BW reduction at week 24 (Table S4). Elevated serum levels of ALP were associated with endothelial dysfunction in hypertensive individuals.^24^ Also, it was experimentally suggested that the robust induction of vascular ALP activity by oxidative stress was related to vascular calcification.^25^ Elevated levels of ALP were shown to interact with an oxidative stress‐mediated elevation in GGT and were related to mortality risk in patients with chronic kidney disease.^26^ These findings suggest an association between ALP and GGT through oxidative stress. On that basis, we speculate that tofogliflozin reduces ALP levels mainly via its anti‐hyperglycaemic effects leading to the reduction in hyperglycaemia‐mediated oxidative stress. Further investigations will be needed because there are several ALP isozymes to be considered. Indeed, in the present study, the time‐course of BAP change was similar to the ALP reduction.

ALT is detected primarily in the liver whereas GGT is also found in other organs. Therefore, ALT may reflect hepatic events more specifically than GGT while GGT may reflect general oxidative stress. In addition, serum ALT levels are directly correlated with hepatic fat accumulation, with decreases accompanying reductions in liver fat.^5^ In agreement with these findings, the time course of ALT reductions was similar to that of weight loss during the tofogliflozin intervention in the present study.

It was reported that half‐lives of GGT and ALT are approximately 7–10 days and 47 h, respectively, in humans.^27^, ^28^ Therefore, differences in half‐lives alone do not explain distinct modes of reductions in GGT and ALT in the present study because GGT has a longer half‐life than ALT.

We previously evaluated the histological course of NAFLD in Japanese patients and found that a reduction in HbA1c levels predicted regression of liver fibrosis, suggesting that strict glycemic control might ameliorate liver fibrosis.^4^ In addition, lifestyle intervention‐induced weight loss was indicated to improve liver histology in patients with NASH.^29^, ^30^ In this regard, our current results showed that in T2D patients both GGT and ALT levels were reduced during the intervention with tofogliflozin in association with respective reductions in glycemic indicators and BW. Therefore, SGLT2 inhibitors could be expected to ameliorate NAFLD through their anti‐diabetic and anti‐obesity effects.

The present study has some limitations. First, we could not completely separate the effects of reductions in glucose and BW because tofogliflozin reduced values for both. Therefore, we focused on distinct differences in the time‐course of reductions in parameters after initiating tofogliflozin treatment. Meta‐analyses of the effects of various agents that reduce glucose without reducing BW, such as sulfonylurea and insulin, on GGT and ALT may further confirm our hypothesis. Second, endpoints in each clinical trial did not involve evaluations of the liver such as by ultrasound, fibroscan, MRI, and biopsy. Future studies should consider the association of changes in liver histology with changes in liver enzymes, glycemic status, and obesity. Third, our conclusion was derived from pooled analyses of clinical trials and did not elucidate mechanisms underlying the association of each liver enzyme with hyperglycaemia and obesity. Further study is needed to evaluate surrogate markers for inflammation and oxidative stress, such as circulating levels of inflammatory cytokines and antioxidant enzymes in association with liver enzymes. In addition, single‐cell RNA sequence analyses of the liver will identify responsible cell clusters that produce individual liver enzymes in response to hyperglycaemia and obesity.

In conclusion, reductions in GGT and ALT were associated with the anti‐hyperglycaemic and anti‐obesity effects of tofogliflozin, respectively, in people with T2D. Therefore, the liver enzymes GGT and ALT may be helpful as surrogate markers for the effects of individualized therapy for T2D by improving our understanding of a patient's pathophysiology with respect to hyperglycaemia and obesity. Past and future epidemiological data should be re‐evaluated with reference to this hypothesis. The molecular mechanisms underlying the association of specific liver enzymes with hyperglycaemia and obesity should be investigated in the future.

## AUTHOR CONTRIBUTIONS

**Toshinari Takamura:** Conceptualization (lead); data curation (equal); formal analysis (equal); investigation (lead); project administration (lead); writing – original draft (equal); writing – review and editing (lead). **Kohei Kaku:** Project administration (equal); supervision (equal). **Akihiro Yoshida:** Data curation (equal); investigation (equal); writing – original draft (equal). **Hiromi Kusakabe:** Data curation (supporting); investigation (supporting). **Hiroyuki Nakamura:** Formal analysis (equal); investigation (equal); methodology (equal). **Hideki Suganami:** Formal analysis (lead); investigation (equal); methodology (equal); project administration (equal).

## FUNDING INFORMATION

The original phase 2 and phase 3 studies of tofogliflozin were funded by Chugai Pharmaceutical Co., Ltd.

## CONFLICT OF INTEREST STATEMENT

TT has received honoraria for lectures and scholarship grants from Astellas Pharma, Sanofi, Kowa, AstraZeneca, Ono Pharma, Tanabe Pharma, Novartis, Mitsubishi Tanabe Pharma, Daiichi Sankyo, and Nippon Boehringer Ingelheim Co., Ltd. KK has been an advisor to and received honoraria for lectures from Astellas Pharma, Novo Nordisk Pharma, Sanwa Kagaku Kenkyusho, Takeda, Taisho Pharmaceutical, MSD, Kowa, Kissei, Sumitomo Dainippon Pharma, Novartis, Mitsubishi Tanabe Pharma, Nippon Boehringer Ingelheim, Daiichi Sankyo, and Sanofi. AY, HK and HS are employees of Kowa Co., Ltd.

## Supporting information


**Supplemental Figure 1:** Time‐course percentage changes in levels of BAP (A), and NTx (B)
Observed least square mean (standard error)
● placebo, ● tofogliflozin
The time courses of the percent changes in BAP, and NTx during entire intervention were tested using a mixed effect model with treatment (tofogliflozin vs. placebo) and time (week 12 and 24) and interaction between treatment and time as fixed effects with the baseline value as a covariate, and subject as a random effect. P value for time‐treatment interaction was reported.
Abbreviations: BAP, bone specific alkaline phosphatase; NTx, type I collagen cross‐linked N‐telopeptide

**Supplemental Table 1.** Summary of each study design and main inclusion criteria
Supplemental Table 2. Integrated analysis of four clinical studies of the use of tofogliflozin
Supplemental Table 3. Correlations among baseline fasting plasma glucose, BMI, and hepatic enzymes
Supplemental Table 4. Potential baseline factors influencing baseline GGT and ALT levels
Supplemental Table 5. Correlations among percent changes in fasting plasma glucose, body weight, and hepatic enzymes at week 24 in participants receiving tofogliflozin
Supplemental Table 6. Potential factors influencing the percentage of changes in GGT and ALT levels at week 24 in participants receiving tofogliflozin

## Data Availability

The data that support the findings of this study are available on request from the corresponding author. The data are not publicly available due to privacy or ethical restrictions.
