# Supplementary material for: Reductions in liver enzymes are associated with anti‐hyperglycaemic and anti‐obesity effects of tofogliflozin in people with type 2 diabetes: Post‐hoc analyses
Source: Endocrinol Diabetes Metab. 2023 Nov 20;7(1):e461. doi: 10.1002/edm2.461 (PMC10782046; doi:10.1002/edm2.461)
Supplement: Supplementary file 1 — Supplemental Figure 1: Time‐course percentage changes in levels of BAP (A), and NTx (B) Observed least square mean (standard error) ● placebo, ● tofogliflozin The time courses of the percent changes in BAP, and NTx during entire intervention were tested using a mixed effect model with treatment (tofogliflozin vs. placebo) and time (week 12 and 24) and interaction between treatment and time as fixed effects with the baseline value as a covariate, and subject as a random effect. P value for time‐treatment interaction was reported. Abbreviations: BAP, bone specific alkaline phosphatase; NTx, type I collagen cross‐linked N‐telopeptide [file EDM2-7-e461-s001.pptx]

## Slide 1
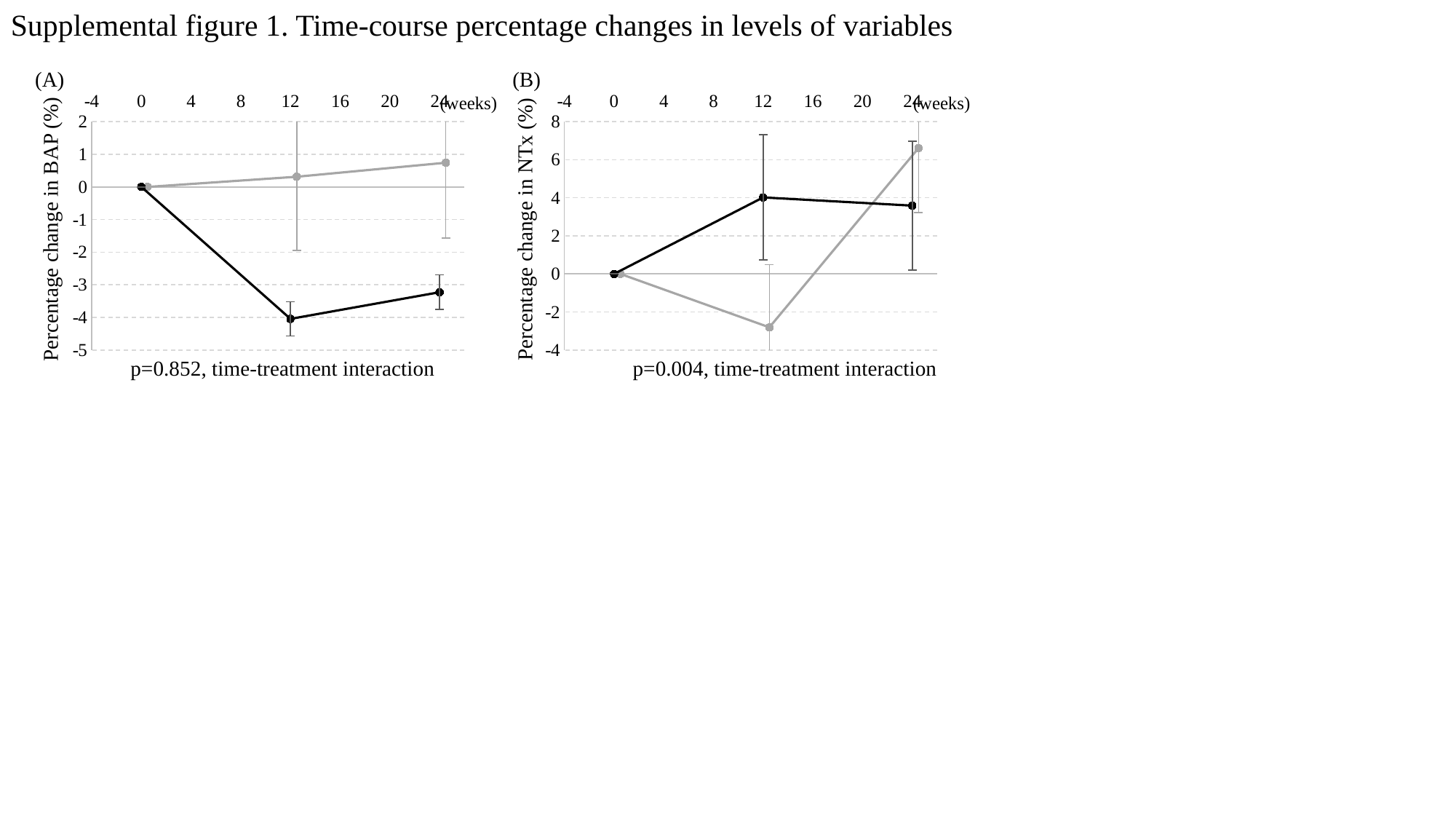

Supplemental figure 1. Time-course percentage changes in levels of variables
(A)
(B)
### Chart
| Category | | |
|---|---|---|(weeks)
### Chart
| Category | | |
|---|---|---|(weeks)
Percentage change in BAP (%)
Percentage change in NTx (%)
p=0.852, time-treatment interaction
p=0.004, time-treatment interaction
