# Supplementary material for: Reductions in liver enzymes are associated with anti‐hyperglycaemic and anti‐obesity effects of tofogliflozin in people with type 2 diabetes: Post‐hoc analyses
Source: Endocrinol Diabetes Metab. 2023 Nov 20;7(1):e461. doi: 10.1002/edm2.461 (PMC10782046; doi:10.1002/edm2.461)
Supplement: Supplementary file 2 — Supplemental Table 1. Summary of each study design and main inclusion criteria Supplemental Table 2. Integrated analysis of four clinical studies of the use of tofogliflozin Supplemental Table 3. Correlations among baseline fasting plasma glucose, BMI, and hepatic enzymes Supplemental Table 4. Potential baseline factors influencing baseline GGT and ALT levels Supplemental Table 5. Correlations among percent changes in fasting plasma glucose, body weight, and hepatic enzymes at week 24 in participants receiving tofogliflozin Supplemental Table 6. Potential factors influencing the percentage of changes in GGT and ALT levels at week 24 in participants receiving tofogliflozin [file EDM2-7-e461-s002.docx]

Supplemental Table 1. Summary of each study design and main inclusion criteria

| Study | Therapy | Design | Duration, weeks | Allocation, n |  |  | Inclusion criteria |  |
| --- | --- | --- | --- | --- | --- | --- | --- | --- |
|  |  |  |  |  | HbA1c,  % | BMI,  kg/m^2^ | sCr, mg/dL  eGFR, mL/min/1.73m^2^ | Age,  years |
| CSG003JP^1^ | Monotherapy | randomized, placebo-controlled, double-blind, parallel-group comparative study | 24 | Placebo (56) TOFO 10 mg (57) TOFO 20 mg (58) TOFO 40 mg (58) | ≧7.3,  ＜10.3 | ≧18.5,  ＜45.0 | Male; sCr＜2.0 Female; sCr＜1.5 | ≧20,  ＜75 |
| CSG004JP^2^ | Monotherapy | open-label, randomized controlled study | 52 | TOFO 20 mg (63) TOFO 40 mg (127) | ≧6.8,  ＜10.3 | ≧18.5,  ＜45.0 | Male; sCr＜2.0 Female; sCr＜1.5 | ≧20 |
| CSG005JP^2^ | Add-on to SU  Add-on to Glinide  Add-on to BG  Add-on to TZD  Add-on to α-GI  Add-on to DPP-4i | open-label, randomized controlled study | 52 | TOFO 20 mg (172) TOFO 40 mg (413) | ≧6.8,  ＜10.3 | ≧18.5,  ＜45.0 | Male; sCr＜2.0 Female; sCr＜1.5 | ≧20 |
| CSG006JP^3^ | Monotherapy  Add-on to SU  Add-on to DPP-4i | open-label, prospective study | 24 | TOFO 40 mg (42) | ≧6.8,  ＜10.3 | ≧18.5,  ＜45.0 | 30.0≦eGFR＜60.0  or 90≦eGFR | ≧20,  ＜75 |

Abbreviations: HbA1c, glycosylated hemoglobin; BMI, body mass index; eGFR, estimated glomerular filtration rate; TOFO, tofogliflozin; SU, sulfonylurea; BG, biguanide; TZD, thiazolidine; α-GI, α-glucosidase inhibitor; DPP-4i, dipeptidyl peptidase-4 inhibitor.

1 Kaku K et al. Cardiovasc Diabetol. 2014 Mar 28;13:65.

2 Tanizawa Y et al. Expert Opin Pharmacother. 2014 Apr;15(6):749-66.

3 Sachiya Ikeda et al. Drug Res (Stuttg) . 2019 Jun;69(6):314-322.

Supplemental Table 2. Integrated analysis of four clinical studies of the use of tofogliflozin

| Study | TOFO Dosage (n) | Periods (weeks) |  |  |  | Baseline |  |  |  |  |
| --- | --- | --- | --- | --- | --- | --- | --- | --- | --- | --- |
|  |  |  | Sex  (Men/Women) | Age (years) | BMI (kg/m^2^) | HbA1c (%) | HbA1c  (mmol/mol) | ALT (IU/L) | GGT (IU/L) | eGFR (ml/min/1.73m^2^) |
| CSG003JP^1^ | Placebo (56) TOFO 10 mg (57) TOFO 20 mg (58) TOFO 40 mg (58) | 24 | 153 / 76 | 57.3 (9.7) | 25.5 (4.1) | 8.4  (0.8) | 68.2  (8.4) | 29.3 (17.9) | 50.7  (63.0) | 85.4  (18.8) |
| CSG004JP^2^ | TOFO 20 mg (63) TOFO 40 mg (127) | 52 | 126 / 64 | 58.1 (10.8) | 25.6 (4.5) | 7.8  (0.9) | 62.1  (9.9) | 29.8 (16.7) | 44.7  (34.5) | 83.0  (18.2) |
| CSG005JP^2^ | TOFO 20 mg (172) TOFO 40 mg (413) | 52 | 386 / 199 | 58.6 (10.4) | 25.6 (4.3) | 8.1  (0.9) | 65.3  (9.8) | 29.2 (15.9) | 47.3  (50.3) | 84.2  (18.4) |
| CSG006JP^3^ | TOFO 40 mg (42) | 24 | 35 / 7 | 61.5 (9.9) | 25.8 (3.3) | 7.8  (1.0) | 61.8  (10.5) | 25.9 (17.1) | 55.3  (52.3) | 65.3  (28.8) |
| Total | Placebo (56)  TOFO 10 mg (57)  TOFO 20 mg (293)  TOFO 40 mg (640) | 24 | 700 / 346 | 58.3 (10.3) | 25.6 (4.2) | 8.1  (0.9) | 65.2  (9.8) | 29.2 (16.5) | 47.9  (51.1) | 83.5  (19.3) |

Data are expressed as mean (standard deviation).

Abbreviations: ALT, alanine aminotransferase; GGT, gamma-glutamyltransferase.

1 Kaku K et al. Cardiovasc Diabetol. 2014 Mar 28;13:65.

2 Tanizawa Y et al. Expert Opin Pharmacother. 2014 Apr;15(6):749-66.

3 Sachiya Ikeda et al. Drug Res (Stuttg). 2019 Jun;69(6):314-322.

Supplemental Table 3. Correlations among baseline fasting plasma glucose, BMI, and hepatic enzymes

| Factors | FPG (mg/dL) | | BMI (kg/m^2^) | |
| --- | --- | --- | --- | --- |
|  | Rho | p | Rho | p |
| GGT (IU/L) | 0.24 | <0.001 | 0.24 | <0.001 |
| ALT (IU/L) | 0.14 | <0.001 | 0.40 | <0.001 |
| ALP (IU/L) | 0.16 | <0.001 | 0.004 | 0.90 |
| AST (IU/L) | -0.03 | 0.28 | 0.20 | <0.001 |

Correlation analysis was performed using Spearmanʼs correlation coefficient

Abbreviations: ALP, alkaline phosphatase

Supplemental Table 4. Potential baseline factors influencing baseline GGT and ALT levels

| GGT levels |  |  |
| --- | --- | --- |
| Factors | Regression Coefficient | P |
| AST (>1 IU/L) | 1.95 | <0.001 |
| FPG (> 10 mg/dL) | 1.90 | <0.001 |
| eGFR (> 1 ml/min/1.73m^2^) | 0.14 | 0.054 |
|  |  |  |
| ALT levels |  |  |
| Factors | Regression Coefficient | P |
| AST (>1 IU/L) | 1.15 | <0.001 |
| BMI (>1 kg/m^2^) | 0.37 | <0.001 |
| HbA1c (>0.1 %) | 0.19 | <0.001 |
| HOMA-β (>1 unit) | 0.03 | <0.001 |
| Duration of diabetes (>1 year) | -0.14 | 0.004 |
| Age (>1 year) | -0.24 | <0.001 |

Stepwise variable selection was used for the analyses.

Adjusted for age, sex, duration of diabetes, HbA1c, FPG, HOMA-IR, HOMA-β, BMI, eGFR, waist circumference, AST, ALT, GGT

Supplemental Table 5. Correlations among percent changes in fasting plasma glucose, body weight, and hepatic enzymes at week 24 in participants receiving tofogliflozin

| Factors | FPG (%) | | Body weight (%) | |
| --- | --- | --- | --- | --- |
|  | Rho | p | Rho | p |
| GGT (%) | 0.23 | <0.001 | 0.26 | <0.001 |
| ALP (%) | 0.28 | <0.001 | 0.05 | 0.13 |
| ALT (%) | 0.17 | <0.001 | 0.20 | <0.001 |
| AST (%) | 0.03 | 0.33 | 0.14 | <0.001 |

Correlation analysis was performed using Spearmanʼs correlation coefficient

Supplemental Table 6. Potential factors influencing the percentage of changes in GGT and ALT levels at week 24 in participants receiving tofogliflozin

| Percent change in GGT levels at week 24 |  |  |
| --- | --- | --- |
| Factors | Regression Coefficient | P |
| Percent change in body weight at week 24 (>1 %) | 1.52 | <0.001 |
| Percent change in FPG at week 24 (>1 %) | 0.22 | <0.001 |
| AST (>1 IU/L) | 0.19 | 0.001 |
| Age (>1 year) | 0.11 | 0.001 |
| Percent change in HOMA-IR at week 24 (>1 %) | 0.07 | <0.001 |
| FPG (>1 mg/dL) | 0.03 | 0.011 |
| HOMA-IR (>0.1 unit) | 0.06 | <0.001 |
| GGT (>1 IU/L) | -0.08 | <0.001 |
| ALT (>1 IU/L) | -0.32 | <0.001 |
|  |  |  |
| Percent change in ALT levels at week 24 |  |  |
| Factors | Regression Coefficient | P |
| Percent change in body weight at week 24 (>1%) | 1.09 | <0.001 |
| AST (>1 IU/L) | 0.16 | 0.004 |
| eGFR (>1 ml/min/1.73m^2^) | 0.04 | 0.014 |
| Percent change in HOMA-IR at week 24 (>1 %) | 0.03 | 0.003 |
| ALT (>1 IU/L) | -0.59 | <0.001 |

Stepwise variable selection was used for the analyses.

Adjusted for age, sex, duration of diabetes, HbA1c, FPG, HOMA-IR, HOMA-β, BMI, eGFR, waist circumference, AST, ALT, GGT, the percentage change in FPG at week 24, the percentage change in body weight at week 24 and the percentage change in HOMA-IR at week 24.
